# Salvage re-irradiation using stereotactic body radiation therapy for locally recurrent prostate cancer: the impact of castration sensitivity on treatment outcomes

**DOI:** 10.1186/s13014-021-01839-w

**Published:** 2021-06-23

**Authors:** Ron Lewin, Uri Amit, Menachem Laufer, Raanan Berger, Zohar Dotan, Liran Domachevsky, Tima Davidson, Orith Portnoy, Lev Tsvang, Maoz Ben-Ayun, Ilana Weiss, Zvi Symon

**Affiliations:** 1 Radiation Oncology Department, Sheba Medical Center, 52621 Ramat-Gan, Israel; 2 Institute of Urology, Sheba Medical Center, Ramat-Gan, Israel; 3 Institute of Oncology, Sheba Medical Center, Ramat-Gan, Israel; 4 Sackler School of Medicine, Tel Aviv University, Tel Aviv-Yafo, Israel; 5 Department of Nuclear Medicine, Sheba Medical Center, Ramat-Gan, Israel; 6 Department of Radiology, Sheba Medical Center, Ramat-Gan, Israel

**Keywords:** Prostate, Salvage, Re-irradiation, Stereotactic body radiation therapy, Castrate resistant

## Abstract

**Background:**

Advances in imaging, biomaterials and precision radiotherapy provide new opportunities to salvage locally recurrent prostate cancer (PC). This study evaluates the efficacy and safety of re-irradiation using stereotactic body radiation therapy (SBRT). We hypothesized that patients with castrate-resistant PC (CRPC) would benefit less from local salvage.

**Methods:**

A prospective clinical database was reviewed to extract 30 consecutive patients treated with prostate re-irradiation. Gallium prostate specific membrane antigen (PSMA) ligand positron emission tomography was performed following prostate-specific antigen failure in all patients and biopsy was obtained in 18 patients (60%). Re-irradiation was either focal (n = 13) or whole-gland (n = 17). Endo-rectal balloons were used in twenty-two patients and hydrogel spacers in eight patients. The median prescription dose was 5 fractions of 6.5 (range: 6–8) Gray (Gy).

**Results:**

Median follow-up was 28 months*.* Failure occurred in 10 (out of 11) CRPC patients versus 6 (out of 19) castrate-sensitive patients (91% vs. 32%, *p* = 0.008) after a median of 13 and 23 months, respectively. Metastases occurred in 64% (n = 7) of CRPC patients versus 16% (n = 3) of castrate-sensitive patients (*p* = 0.007). Two patients experienced local in-field recurrence, thus local control was 93%. The 2 and 3-year recurrence-free survival were 84% and 79% for castrate-sensitive patients versus 18% and 9% for CRPC patients (*p* < 0.001), and 3-year metastasis-free survival was 90% versus 27% (*p* < 0.01) for castrate-sensitive and CRPC patients, respectively. Acute grade II and III genitourinary (GU) toxicity occurred in 27% and 3%, and late GU toxicity in 30% and 3%, respectively. No ≥ grade II acute gastrointestinal (GI) toxicity occurred, and only one patient (3%) developed late grade II toxicity.

**Conclusions:**

Early delivery of salvage SBRT for local recurrence is associated with excellent 3-year disease control and acceptable toxicity in the castrate-sensitive phenotype. PSMA imaging for detection of local recurrence and the use of precision radiotherapy with rectal protective devices should be further investigated as a novel salvage strategy for radio-recurrent PC.

## Background

Recurrence of PC after local definitive radiation therapy (RT) is a common clinical scenario. Up to a third of patients undergoing primary RT to the prostate will experience biochemical failure within 10 years [1–3]. The most common pattern of relapse for all risk groups is local recurrence within the prostate or seminal vesicles (SV) [4, 5]. In the absence of distant metastases, salvage local treatment may lead to prolonged disease control and possibly cure. There is no clear consensus on the optimal management of primary RT failure. Acceptable treatment options includes local salvage with radical prostatectomy, cryotherapy, and focal therapies such as high-intensity focused ultrasound (HIFU) [5]. However as these options are often associated with urinary and sexual morbidity, many patients are managed with lifelong androgen deprivation therapy (ADT) [6, 7], which has a major impact on quality of life [8], is not curative by itself, and eventually induces a more aggressive CRPC phenotype [9]. In recent years, re-irradiation using brachytherapy [10–14] has been included in guideline options and SBRT is under investigation [15–17].

Improvements in techniques of imaging to locate sites of recurrence and to guide biopsies or focal therapies have been considerable. PSMA-PET imaging and dynamic contrast enhanced magnetic resonance imaging (DCE-MRI) are useful tools for defining regions suspicious for recurrence [5, 18–21]. Furthermore, considerable advances in precise treatment planning and delivery, as well as the introduction of endo-rectal balloons (ERBs) [22, 23] and spacers [24–26] have improved the ability to offer safer re-irradiation in recurrent disease.

The purpose of this study is to report safety and efficacy outcomes of prostate re-irradiation using SBRT with contemporary planning and delivery techniques. In addition, we aim to stratify treatment outcomes according to castrate sensitivity status for better patient selection. We hypothesized that local salvage of castrate-sensitive versus CRPC patients would be associated with improved outcomes.

## Methods

Patient data was extracted from our Institutional Review Board approved clinical database. Eligible patients were treated with salvage SBRT to the prostate following local failure of primary external-beam RT (EBRT) or brachytherapy. Patients with a history of severe late toxicity, such as radiation proctitis, cystitis, or urinary stricture following previous RT were not eligible for salvage SBRT. Patients were specifically asked about the occurrence and severity of such symptoms, and medical records were also scrutinized to identify events suggestive of grade 3 or 4 toxicity. In questionable cases, rectoscopy and cystoscopy were performed prior to re-irradiation. All patients had experienced a biochemical failure after primary RT defined as a rise in PSA > 2 ng/ml above nadir PSA value according to the Phoenix criteria. In order to study the effects of CRPC phenotype on outcomes, patients receiving salvage SBRT were categorized as harboring castrate-sensitive or castrate-resistant local recurrence. Castrate-sensitive patients had not received androgen ablation for 2 years prior to the recurrence and salvage SBRT. CRPC patients had all received androgen ablation following failure of primary radiation and were not initially referred for consideration of local salvage by their treating physicians. They subsequently developed a biochemical failure on ADT, were find to harbor local disease on imaging and then referred for salvage radiation. Ga^68^ PSMA PET imaging was performed in all patients prior to salvage SBRT, demonstrating at least a local pathological PSMA uptake. Histologic confirmation of local recurrence was the objective in all cases, but not always achieved due to patient or physician preference. All patients treated without a biopsy had very suggestive imaging on both PSMA PET and dynamic contrast-enhanced MRI. Salvage re-irradiation was delivered with SBRT using volumetric modulated arc therapy (VMAT) (RapidArc, Varian medical systems. Palo Alto, Ca). Focal re-irradiation was the preferred strategy to reduce the risk of toxicity. Clinical target volume (CTV) was defined as the PSMA-avid lesion and the DCE-MRI lesion with a 3–5 mm margin for well-defined lateralized recurrences. Whole gland re-treatment was reserved for patients with large tumors crossing or abutting the midline or bilateral recurrences.

**Fig. 1:**
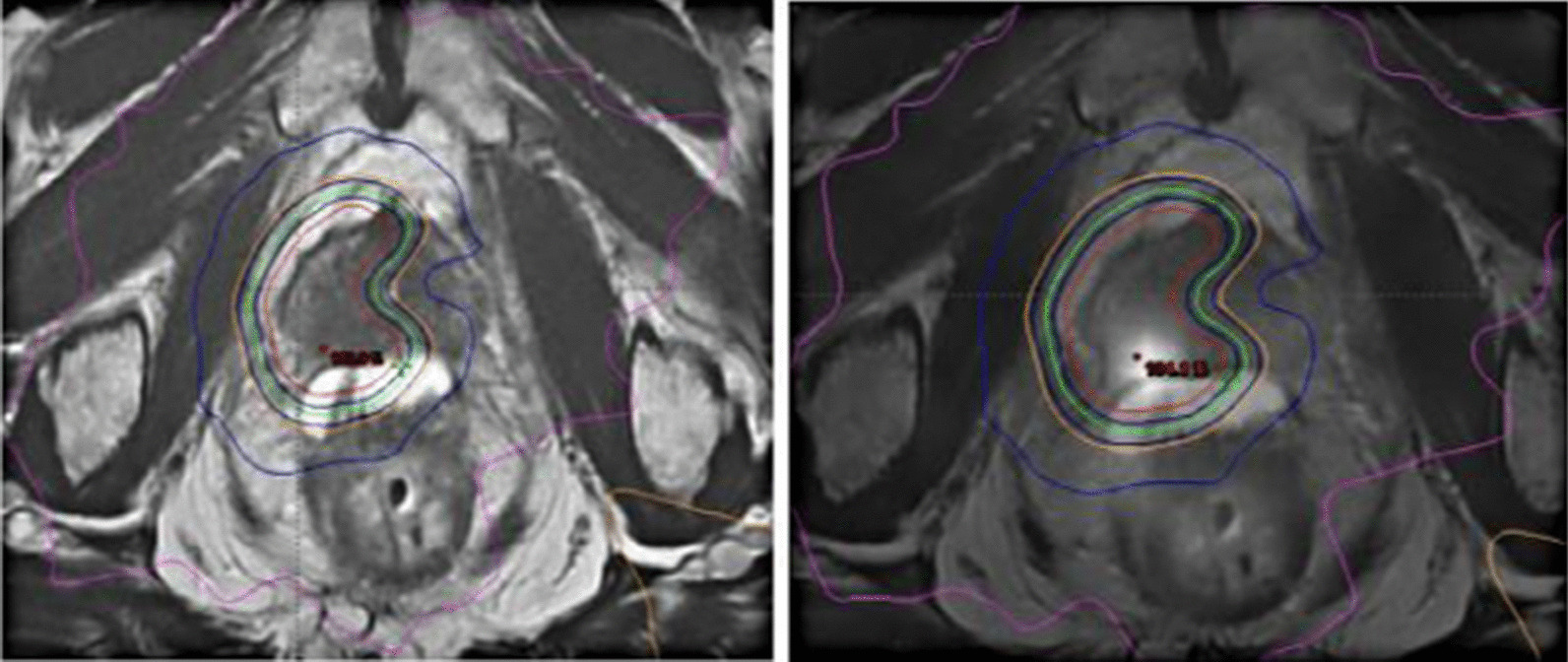
An example of focal re-irradiation using PSMA-PET for target delineation, urethral dose sparing, and a hydrogel rectal spacer

All patients had fiducial markers placed in the prostate. Hydrogel spacers are associated with lower anterior rectal wall dose than ERBs and thus were the preferred method for rectal displacement in patients with recurrence in the prostate gland and were implanted in patients who could afford the cost. ERBs were preferred for seminal vesicle recurrences, as the spacer is not as effective in this setting. Urethral-sparing dose painting was used to reduce urethral toxicity (Fig. 1). For urethra-sparing planning prescribed dose to the urethra planning risk volume (uPRV) was 32.5 Gy (6.5 Gy/fraction), and dose constraints were D98% ≥ 30.875 Gy (95% of 32.5 Gy) and D2% < 35.75 Gy (110% of 32.5 Gy). Dose constraints for the rectal wall were V100% < 5% (of the prescribed dose to the prostate), V90% < 15%, V80% < 20%, and V38 Gy < 2 cc. Treatment was delivered with a full bladder in 5 fractions using image guidance with cone beam computed tomography (CBCT). Toxicity was reported using the common terminology criteria for adverse events (CTCAE) v4.0. Acute toxicity was defined as adverse reactions occurring within 3 months after salvage SBRT, and late toxicity for those occurring later than 3 months.

Median follow-up time was calculated from salvage SBRT till recurrence or death. RFS was defined as the length of time from salvage SBRT to either biochemical/clinical failure or death from prostate cancer, and was estimated using the Kaplan–Meier method. Metastasis-free survival (MFS) was defined as the time from salvage SBRT to the date of onset of new distant metastases or when censored at last date. The log rank test was used to assess the difference between groups (SPSS Inc., IBM). P-value < 0.05 was considered statistically significant. The difference in probability of local in-field recurrence after salvage SBRT between castrate-sensitive and CRPC patients was compared using the chi-square test.

## Results

Thirty patients treated between 2015 and 2018 with re-irradiation to the prostate following local failure were extracted from our database.

### Patient and treatment characteristics at initial diagnosis

The median age at time of primary RT was 62 years (range: 52–75 years). Patients were classified as low (n = 7), intermediate (n = 11), and high (n = 12) risk according to the national comprehensive cancer network (NCCN) risk stratification. Primary RT to the prostate was given either with EBRT (n = 25) to a median dose of 80 Gy (range: 74–82 Gy), or low dose rate (LDR) brachytherapy (n = 5) using I^125^ to a prescription dose of 145 Gy. All high-risk patients and 5 intermediate risk patients received long and short course ADT, respectively, in conjunction with RT.

### Primary RT failure

Median time from primary RT to biochemical/clinical failure was 72 months (range: 18–176 months). One third of patients (n = 11) received ADT-only as first salvage treatment upon failure for a median duration of 53 months (7–191 months) leading to the emergence of CRPC prior to their referral for local salvage treatment. Ga^68^ PSMA PET scans were performed in all patients prior to salvage SBRT*.* All patients had local pathological PSMA uptake: prostate-only in 18 patients, prostate and SV in 10 patients, and SV-only in 2 patients. Local staging at recurrence was stage ≥ T3 in 14 patients (47%). Regional (nodal) pathological uptake was also observed in 7 patients (23%), one of whom had two small vertebral metastases. Biopsy confirming recurrent prostate adenocarcinoma was performed in 18 patients (60%). Gleason score at recurrence was only reported in 8 of these cases, all in the high-risk category (Gleason 10 in one patient, Gleason 9 in six patients, and Gleason 8 in one patient).

### Patient and tumor characteristics prior to salvage SBRT

Median interval from primary RT to re-treatment was 9 years (range 2–20 years). Histologic confirmation of recurrence was obtained in 60% of patients. Patient and tumor characteristics prior to salvage SBRT are detailed in Table 1.

**Table 1:** Patient and tumor characteristics

	All patients (n = 30)	Castrate-resistant (n = 11)	Castrate-sensitive (n = 19)
*Age, years* Median (range)	71 (56–83)	72 (56–83)	69 (57–80)
*Pre-salvage PSA, ng/ml* Median (range)	3.63 (0.05–77)	3.16 (0.05–12.8)	4.1 (0.65–77)
*Pre-salvage imaging, n (%)*			
Ga^68^ PSMA PET	30 (100%)	11 (100%)	19 (100%)
mpMRI	11 (37%)	4 (36%)	7 (37%)
*Location of local recurrence, n (%)*			
Prostate-only	18 (60%)	8 (73%)	10 (53%)
Prostate and SV	10 (33%)	3 (27%)	7 (37%)
SV-only	2 (7%)	0 (0%)	2 (11%)
*Staging at recurrence, n (%)*			
T-stage ≥ T3 (locally advanced)	14 (47%)	6 (55%)	8 (42%)
Node positive (regional)	7 (23%)	3 (27%)	4 (21%)
Oligo-metastatic	1 (3%)	1 (9%)	0 (0%)
*Histologic confirmation, n (%)*			
Yes	18 (60%)	6 (55%)	12 (63%)
Median Gleason score^1^ (range)	9 (8–10)		

Note that 83% of patients had high-risk features at recurrence (either castrate-resistant, Gleason ≥ 8, T-stage ≥ T3, or node positive)

SBRT, stereotactic body radiation therapy; PSA, prostate specific antigen; mpMRI, multi-parametric magnetic resonance imaging; Ga^68^-PSMA, gallium-68 prostate specific membrane antigen positron; PET, positron emission tomography; SV, seminal vesicles

^1^Data was available for 8 patients

The median salvage SBRT dose was 5 fractions of 6.5 Gy (range: 6–8 Gy) given every other day. Whole-gland salvage SBRT was given in 17 patients (57%) and focal salvage therapy in 13 (43%) patients. Urethral sparing was achieved in 17 (57%) patients (in 13 patients by focal therapy and in 4 receiving whole gland). Curative attempt radiotherapy was delivered to all disease sites in patients with PSMA uptake in pelvic nodes and in patients with oligometastases. Thus one patient with 2 oligo-metastases received SBRT to the vertebral lesions to a dose of 24 Gy in 3 fractions in addition to prostate re-irradiation. Seven patients (23%) with PSMA-avid lymph nodes also received nodal SBRT to a total dose of 25 Gy in 5 fractions. Rectal sparing techniques were utilized in all patients, ERB (Rectal Pro75, ELRAD International Ltd, International Warehouse, Dam Sluisweg 6, 1332 EC Almere) in 22 (73%) patients, and hydrogel spacer (Boston Scientific Corp., Marlborough, Massachusetts) in 8 (27%) patients. Short-term ADT was initiated with salvage SBRT in 15 out of 19 (79%) patients with castrate-sensitive disease, while additional 11 CRPC patients continued long-term ADT concomitantly with salvage SBRT. Treatment characteristics are shown in Table 2.

**Table 2:** Treatment characteristics

	All patients (n = 30)	Castrate-resistant (n = 11)	Castrate-sensitive (n = 19)
*Target volume*			
Whole gland therapy	17 (57%)	11 (100%)	6 (32%)
Focal salvage therapy	13 (43%)	0 (0%)	13 (68%)
*LHRH agonist*			
Initiated with salvage SBRT (for 6 months)	15 (50%)		15 (79%)
Continued with salvage SBRT	11 (100%)	11 (100%)	
*2nd generation anti-androgen*			
Initiated with salvage SBRT			1 (5%)
Continued with salvage SBRT^1^		1 (9%)	
*Rectal sparing*			
Endo-rectal balloon	22 (73%)	7 (64%)	15 (79%)
Hydrogel spacer	8 (27%)	4 (36%)	4 (21%)
*Urethral sparing*			
Yes	17 (57%)	3 (27%)	14 (74%)
*Prostate dose/fraction (Gy)*			
Median (range)	6.5 (6–8)	6.25 (6–7.25)	6.5 (6–8)
Number of fractions	5	5	5
*Pelvic lymph nodes irradiation*			
Given in^2^	7 (23%)	3 (27%)	5 (26%)

SBRT, salvage stereotactic body radiation therapy; Gy, gray; LHRH, luteinizing hormone-releasing hormone

^1^Castrate-resistant patients

^2^Dose for nodal SBRT was 25 Gy in 5 fractions

### Treatment outcomes and toxicities

Median follow-up following salvage SBRT was 28 months (range: 19–83 months)*.* Biochemical / clinical failure post salvage SBRT occurred in 16 patients (53%) after a median time of 16 months (range: 6–38 months). Ten of these patients (63%) developed metastatic progression (bone and/or viscera), 3 had regional (nodal) failure, and 3 local failures (of which 2 were in-field recurrences). Thus, local control of salvage SBRT to the prostate for the entire cohort was 93%. In subgroup analysis, failure occurred in 10 out of 11 CRPC patients versus 6 out of 19 castrate-sensitive patients (91% vs. 32%), *p* = 0.008*.* Median time to failure in the CRPC group was 13 months (range: 6–28 months) compared to 23 months (range: 10–38 months) in the castrate-sensitive group. Metastatic progression occurred in 7 out of 11 CRPC patients and in 3 out of 19 castrate-sensitive patients (64% vs. 16%, *p* = 0.007).

**Fig. 2:**
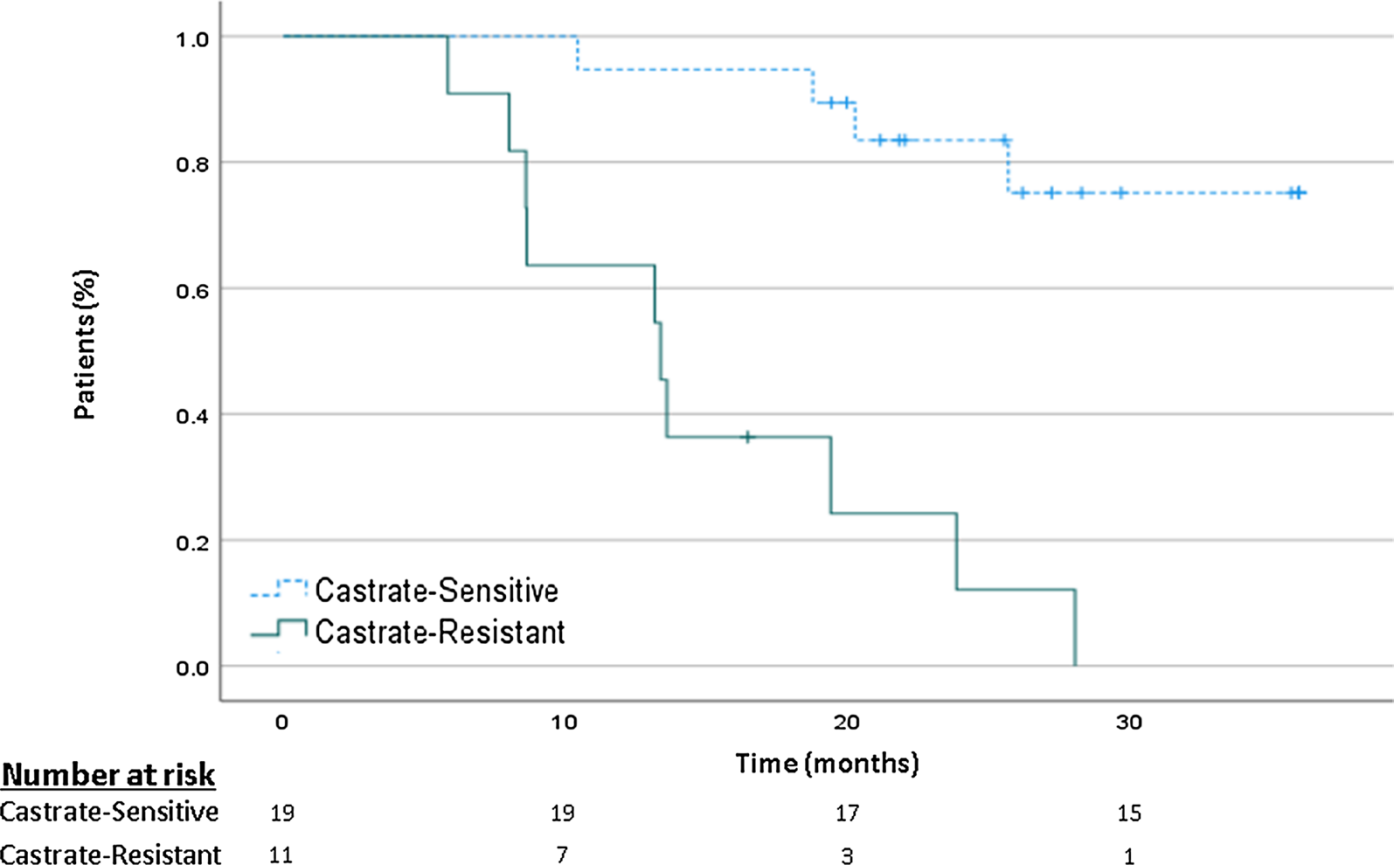
Recurrence-free survival at 3 years

**Fig. 3:**
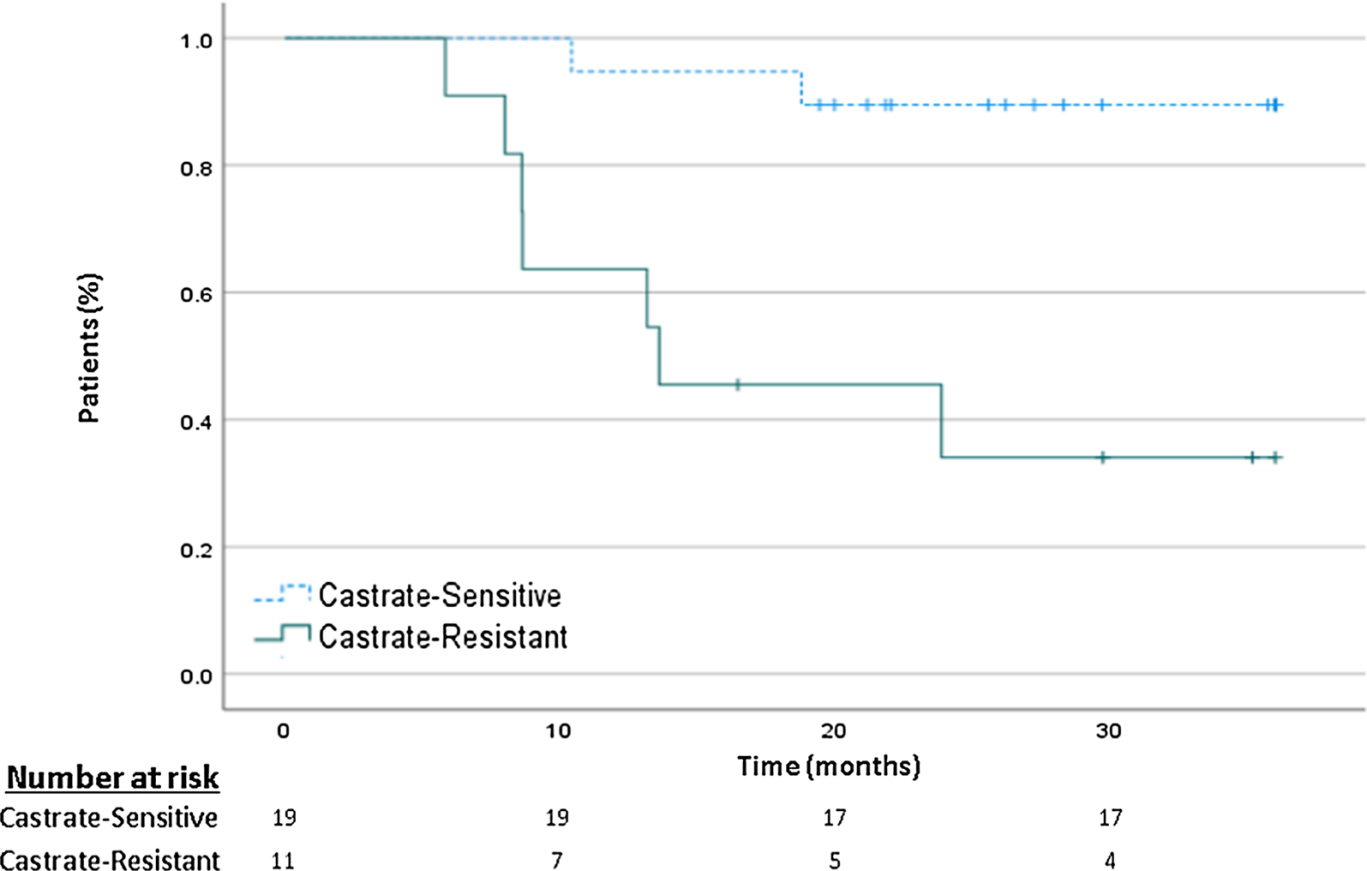
Metastasis-free survival at 3 years

Five out of the 16 patients with disease recurrence had eventually died of their disease after a median interval of 28 months from salvage SBRT. Thirteen patients (68%) in the castrate-sensitive group versus 1 patient (9%) in the CRPC group were without evidence of disease after a median follow-up of 26 months (range: 16–43 months). Median RFS for the entire cohort was 22 months (range: 6–43 months). For castrate-sensitive versus CRPC patients, median RFS was 26 (range: 10–43) versus 13 months (range: 6–28) months, *p* < 0.001. The 2 and 3-year RFS for all patients were 60% and 53%, with a significant difference between castrate-sensitive patients (84% and 79%, respectively) versus CRPC patients (18% and 9%, respectively), *p* < 0.001 (Fig. 2)*.* Median MFS for the entire cohort was 25 months (range: 6–64 months). The 2 and 3-year MFS for the castrate-sensitive group versus the CRPC group were 90% versus 36% (*p* = 0.001) at 2 years, and 90% versus 27% (*p* < 0.001) at 3 years (Fig. 3).

Treatment outcomes are shown in Table 3.

**Table 3:** Treatment outcomes

	All patients (n = 30)	Castrate-resistant (n = 11)	Castrate-sensitive (n = 19)	*p*-value
Biochemical/clinical failure	16 (53%)	10 (91%)	6 (32%)	0.008
Metastatic progression	10 (63%)	7 (64%)	3 (16%)	0.007
Median time to failure, months (range)	16 (6–38)	13 (6–28)	23 (10–38)	
Median RFS, months (range)	22 (6–43)	13 (6–28)	26 (10–43)	< 0.001
2 and 3-year RFS	60% and 53%	18% and 9%	84% and 79%	< 0.001
2 and 3-year MFS		36% and 27%	90% and 90%	0.001

RFS, recurrence-free survival; MFS, metastasis-free survival

Acute grade 2 GU toxicity, primarily cystitis with frequency, urgency, and dysuria occurred in 8 patients (27%), typically commenced mid-treatment and peaked during the first month after treatment and resolved in 3 patients by 3 months, yet persisted as late grade 2 GU toxicity in 5 patients. An additional 4 patients with hardly any early symptoms developed new onset late grade 2 cystitis (n = 2) and incontinence (n = 2), starting 7–9 months after treatment. Thus, late grade 2 GU toxicity occurred in 9 patients (30%). Acute grade 3 hematuria occurred in 1 patient (3%) two months after completion of radiotherapy and required hospitalization and hemostasis, and 1 patient (3%) developed a late grade 3 urethral stricture 10 months after completion of treatment. There was no ≥ grade 2 acute GI toxicity. Late grade 2 GI incontinence occurred in 1 (3%) patient who had suffered from grade 1 fecal incontinence prior to salvage SBRT. No late grade 3 GI events occurred. One patient developed late grade 3 osteomyelitis of the symphysis pubis 9 months following treatment.

## Discussion

In this study we found that for patients with local failure after primary RT to the prostate, re-irradiation with salvage SBRT using rectal protection was associated with excellent local control (93%) and negligible rectal toxicity. For patients with castrate-sensitive local recurrence, salvage SBRT was associated with a 3-year RFS of 80%, suggesting a potential for long-term disease control and possibly cure in these patients. Despite excellent local control, most patients with castrate-resistant local recurrence rapidly developed distant metastases. Overall, about a half of our cohort were relapse free at the time of follow-up without androgen ablation. This was achieved despite the fact that most patients (83%) had high-risk features at recurrence (either CRPC, T3/4 disease, regional nodal spread, or Gleason ≥ 8). Treatment was well-tolerated with an acceptable rate of genitourinary toxicity and no severe rectal toxicity. We hypothesize this was achieved by meticulous treatment preparation, planning and delivery. This includes rectal protection by spacers or endo-rectal balloons, urethral sparing, image-guidance using fiducial markers, and PET/MRI–CT simulation fusions for accurate contouring and smaller margins.

Timing of salvage SBRT and the status of hormonal sensitivity seem to be crucial factors for success. Patients that were initially treated with systemic ADT at first recurrence and subsequently developed castrate-resistant disease prior to salvage SBRT were more prone to rapid development of distant metastases and had significantly poorer outcomes. These patients are presumed to harbor undetectable resistant micro-metastases at time of salvage SBRT with rapid progression to overt metastatic disease following treatment. We believe that salvage therapy should be initiated early in face of local failure, and implementation of novel imaging such as PSMA-PET scans at biochemical failure can improve early detection of local recurrence amendable to local salvage [27]. Early salvage can prevent the emergence of castrate-resistant tumor cells within the prostate, reduce the likelihood of developing distant metastases, improve overall disease control, and potentially can enhance cure rates. If castrate-resistant disease has already emerged then systemic chemotherapy or second-generation anti-androgens should be considered in conjunction with salvage SBRT in order to achieve eradication of such resistant micro-metastatic cells, but this needs to be proven in future trials. A similar disparity in the prognosis of castrate-sensitive versus castrate-resistant patients was shown by Vargas et al. [12] that evaluated salvage brachytherapy in patients with local recurrence following EBRT to the prostate. Five-year biochemical control was 74% for castrate-sensitive and 22% for castrate-resistant cases. Pinkawa et al. had previously reported on results of local prostate RT in patients presenting with PSA progression during primary hormonal treatment. The hazard ratio for failure was 7 for patients with CRPC versus those with castrate sensitive disease [28].

Currently, no clear consensus on the optimal management of primary RT failure exists. Salvage radical prostatectomy can be curative in up to 50% of cases [29–31], but is technically more challenging then primary surgery with higher rates of post-operative complications [31–33], possibly due to radiation-induced fibrosis/adhesions and poorer blood supply and wound healing. Only a small percentage of patients (estimated around 2–3%) with primary RT failure will undergo salvage surgery, and most patients with biochemical recurrence following primary RT are in-fact managed with indefinite ADT monotherapy [4, 5]. Among the alternative strategies for local salvage of radio-recurrent prostate cancer, the most commonly used method is cryotherapy. Third-generation cryotherapy devices have better accuracy and are employed for focal tumor ablation and thus cause less GI/GU morbidity compared to earlier versions [34]. Randomized controlled studies evaluating the different alternatives for local salvage are lacking and there are no standardized criteria for patient selection. Systematic reviews on local salvage modalities suggest similar efficacy (roughly 50% long-term biochemical control) with rates of significant toxicity (albeit with differing side-effect profiles) [35, 36]. In a recently published retrospective trial [37] evaluating salvage cryoablation for radio-recurrent prostate cancer approximately 50% of the patients achieved biochemical salvage with cryoablation at 5 years. Severe complications rates were 3% for rectal fistula, 7% for urethral stricture, 25% for urinary incontinence, and 1% for death secondary to pulmonary embolism. Ng et al. [38] assessed the efficacy of salvage cryoablation in 187 patients with locally recurrent prostate cancer after radiotherapy. Patients with pre-cryoablation PSA < 4 ng/ml had a 5 and 8-year biochemical RFS of 56% and 37%, respectively, and patients with pre-cryoablation PSA ≥ 10 ng/ml had a 5 and 8-year biochemical recurrence-free survival of only 14% and 7%, respectively. Ten percent of patients had persistent lower urinary tract symptoms, 3% severe incontinence and 2% urethra-rectal fistulas.

Our results are in correspondence with other studies evaluating the role of salvage SBRT for local recurrence after radiation therapy, both in efficacy and toxicity. This is in spite of the fact that patients in our cohort had a much higher risk profile then all previously published studies. For the castrate-sensitive subgroup of our study efficacy was much higher. The skeptic who asks “why use radiation again if it failed first time round?” may be offered an answer based on radiobiological modelling suggesting that ultra-hypofractionation is associated with a different mechanism of action and thus may be more effective.

Leroy et al. [15] retrospectively evaluated salvage SBRT for local recurrence following primary RT in 23 patients. SBRT dose was 36 Gy in 6 fractions. Short-term ADT was initiated in 10 patients, and 4 patients were already on ADT prior to salvage SBRT for biochemical failure. Median follow-up was 22.6 months. Two-year disease-free survival (DFS) was 54% and median DFS was 27 months. Local DFS at 1- and 2-years were 100% and 76%. Grade 3 GU cystitis occurred in 2 (9%) patients and 1 patient had grade 3 neuralgia. Cumulative grade 2 GU toxicity occurred in 7 patients (30%), and grade 2 proctitis in 2 (9%) patients.

Pasquier et al. [16] also retrospectively evaluated salvage SBRT for local recurrence after radiation therapy in 100 patients. Median SBRT dose was 36 Gy in 6 fractions. About a third received ADT with SBRT for a median duration of 12 months. Median follow-up was 29 months. Biochemical RFS at 3 years was 55%. The actuarial 3-year grade ≥ 2 GU and GI toxicity was 21% and 1%.

In a study by Fuller et al. 50 patients with local recurrence after prior RT received salvage SBRT [17]. Median follow-up was 44 months. Gleason score at recurrence was 6 (n = 9), 7 (n = 22), 8 (n = 10), and 9 (n = 8). SBRT dose was 34 Gy in 5 fractions. ADT was initiated in 4 patients, and 3 were already on long-term ADT (CRPC). The actuarial 2- and 5-year biochemical RFS rates were 76% and 60%. The 5-year actuarial grade ≥ 2 and grade ≥ 3 GU toxicities were 17% and 8%, respectively. No acute or late GI toxicity grade > 1 occurred.

Our study has several limitations. First, the cohort is relatively small and though we have treated over 60 patients to date, we confined the analysis to patients who had at least 2 years of meticulous follow-up. Despite the relatively modest cohort, the difference between castrate-sensitive and castrate-resistant patients was highly significant, thus generating the hypothesis for more stringent exclusion criteria for aggressive local salvage treatment. About a third of our study cohort had long-standing castrate-resistant disease and most of these patients experienced early distant failure after salvage SBRT. Thus, this study provides some important insights for the success of salvage SBRT including appropriate patient selection and timing of treatment. Secondly, only about two-thirds of patients underwent biopsy for histologic confirmation of local recurrence. In the ideal study all patients should undergo such confirmatory biopsy, however, all patients in our cohort underwent Ga68 PSMA-PET demonstrating local pathologic uptake of PSMA, the positive predictive value (PPV) of which is 99% in the setting of biochemical recurrence (based on pathologic correlation) according to a recent meta-analysis [39]. The strengths of this study include the availability of PSMA-PET and multi-parametric MRI for early detection of local recurrence. Patient follow-up was meticulous with no patients lost to follow-up. The utilization of ERBs and spacers for rectal sparing, precise image guidance using fiducial markers and central urethral sparing were all important measures to minimize toxicity. Rectal toxicity, specifically bleeding, is a major concern for prostate re-irradiation. To decrease the risk of late rectal toxicity, rectal separation was used all patients. The Hydrogel spacer was inserted between the rectum and prostate before RT to create a temporary anatomic separation [40], and was shown to decrease the rate of late rectal toxicity after RT to the prostate. A recent study performed on a MRI–Linac also demonstrated a reduction of rotational shifts and intra-fraction motion with the use of a hydrogel spacer [26]. At 3-year follow-up, all grades of rectal bleeding were decreased in patients with hydrogel spacer. ERBs were shown to immobilize the prostate during RT, thereby allowing for reduced treatment margins [41]. In addition, the use of ERBs significantly reduces rectal dose [23] and severe late rectal toxicity [42].

In order to fully evaluate the true potential benefit of salvage SBRT and its impact on the natural history of the disease, future studies should have a more restrictive patient selection. Eligible patients for local salvage attempt should be patients experiencing biochemical failure following primary RT to the prostate, are referred early for advanced imaging such as PSMA-PET demonstrating pathologic local PSMA uptake, which should be confirmed by biopsy, and time from failure to start of salvage SBRT should be as short as possible (early salvage). The use of concomitant ADT with salvage SBRT is an area of debate. Cuccia et al. [43] have reported a series of salvage SBRT in which the majority of patients did not receive ADT with good local control and the benefit of delaying negative impact on quality of life due to castration. We chose to offer a short course of ADT based on a number of assumptions: (a) Androgen depleted cancer cells are more radiosensitive [44]; (b) The addition of ADT to radiotherapy improves PFS and DMFS in recurrent prostate cancer following surgery [44]; (c) A short duration of ADT is associated with a high incidence of testosterone recovery in the vast majority of men [45]; (d) 83% of our cohort had high risk disease features at recurrence. Duration of concurrent ADT in the setting of salvage SBRT is also an area of debate. Future studies should try to answer whether longer durations of treatment (such as 12 months given in the study by Pasquier [16]) are superior to 6 months of therapy. A recent survey of the Italian Association of Radiotherapy and Clinical Oncology suggests interest in re-irradiation and highlights issues of debate including the irradiated volume, dosimetry parameters, and the use of ADT [46].

## Conclusions

In summary, early delivery of salvage SBRT for local recurrence is associated with excellent 3-year disease control and acceptable toxicity in patients with a castrate-sensitive phenotype. These results appear at the very least comparable to those reported with salvage cryotherapy. Advanced technology for the planning and delivery of salvage re-irradiation with novel measures such as rectal spacers and dose painting all contribute to a low rate of serious treatment-related adverse events.

## Data Availability

The datasets used and/or analyzed during the current study are available from the corresponding author on reasonable request.

## Abbreviations

PC: Prostate cancer
SBRT: Stereotactic body radiation therapy
CRPC: Castrate-resistant prostate cancer
PET: Positron emission tomography
PSMA: Prostate specific membrane antigen
PSA: Prostate-specific antigen
Gy: Gray
RFS: Recurrence-free survival
MFS: Metastasis-free survival
GU: Genitourinary
GI: Gastrointestinal
RT: Radiation therapy
SV: Seminal vesicles
HIFU: High-intensity focused ultrasound
ADT: Androgen deprivation therapy
DCE-MRI: Dynamic contrast enhanced magnetic resonance imaging
ERBs: Endo-rectal balloons
EBRT: External-beam radiation therapy
VMAT: Volumetric modulated arc therapy
CTV: Clinical target volume
uPRV: Urethra planning risk volume
CBCT: Cone beam computed tomography
CTCAE: Common terminology criteria for adverse events
NCCN: National comprehensive cancer network
LDR: Low dose rate
